# Beyond nutrition and physical activity: food industry shaping of the very principles of scientific integrity

**DOI:** 10.1186/s12992-021-00689-1

**Published:** 2021-04-20

**Authors:** Mélissa Mialon, Matthew Ho, Angela Carriedo, Gary Ruskin, Eric Crosbie

**Affiliations:** 1 Trinity Business School, Trinity College Dublin, Dublin, Ireland; 2 School of Community Health Sciences, University of Nevada, Reno, USA; 3 World Public Health and Nutrition Association, London, UK; 4 U.S. Right to Know, Oakland, USA; 5 Ozmen Institute for Global Studies, University of Nevada, Reno, USA

**Keywords:** Conflitct of interest, Commercial determinants of health, Ethics, Food industry, Corporate political activity

## Abstract

**Background:**

There is evidence that food industry actors try to shape science on nutrition and physical activity. But they are also involved in influencing the principles of scientific integrity. Our research objective was to study the extent of that involvement, with a case study of ILSI as a key actor in that space. We conducted a qualitative document analysis, triangulating data from an existing scoping review, publicly available information, internal industry documents, and existing freedom of information requests.

**Results:**

Food companies have joined forces through ILSI to shape the development of scientific integrity principles. These activities started in 2007, in direct response to the growing criticism of the food industry’s funding of research. ILSI first built a niche literature on COI in food science and nutrition at the individual and study levels. Because the literature was scarce on that topic, these publications were used and cited in ILSI’s and others’ further work on COI, scientific integrity, and PPP, beyond the fields of nutrition and food science. In the past few years, ILSI started to shape the very principles of scientific integrity then and to propose that government agencies, professional associations, non-for-profits, and others, adopt these principles. In the process, ILSI built a reputation in the scientific integrity space. ILSI’s work on scientific integrity ignores the risks of accepting corporate funding and fails to provide guidelines to protect from these risks.

**Conclusions:**

The activities developed by ILSI on scientific integrity principles are part of a broader set of political practices of industry actors to influence public health policy, research, and practice. It is important to learn about and counter these practices as they risk shaping scientific standards to suit the industry’s interests rather than public health ones.

**Supplementary Information:**

The online version contains supplementary material available at 10.1186/s12992-021-00689-1.

## Background

Actors in the tobacco, alcohol, and ultra-processed food industries use a broad range of political strategies to protect and expand their markets [1–4]. These practices include direct influence on public health policy, and more subtle actions like cultivating support from communities and the media [1–4]. The shaping of science is one of these political practices [5–8], as science can be used to influence policy [9–11]. Studies that link the consumption of harmful products to ill-health, or those which provide evidence on the effectiveness of a policy that limits consumption, are systematically questioned, attacked, or undermined by companies and third parties working on their behalf [5–8]. Industry actors are also shaping the research agenda by funding commercially-driven science (research supported by the industry) to support their products or practices [12].

When evidence emerged about cigarette smoking’s harmfulness in the 1960s, tobacco companies mounted an attack on science to bury that evidence [13]. However, the tobacco industry understood that it could not credibly question scientific evidence criticizing its products. In the 1980s and 1990s, tobacco companies developed a “*sound science*” program, hiring respected academics and scientists and using third parties to deny secondhand smoke’s harmful effects [14, 15]. Through this program, tobacco companies intended to shape scientific proof standards so that no study could prove that secondhand smoking was harmful [14, 15]. In response, in 2003, the World Health Organization adopted a Framework Convention on Tobacco Control, in which Article 5.3 insulated public health policymaking from the tobacco industry [16]. Although the implementation of Article 5.3 is successful in some contexts [17] and could serve as a model for other industries [18], the tobacco industry is still able to participate in the development of principles for using scientific evidence in policy along with academics and government officials [19].

Similar to the tobacco industry, the food industry also shapes science, through the funding and dissemination of research and information serving its interests and criticizes evidence that may thwart these interests [3, 12, 20]. The food industry established and funded scientific-sounding groups such as the International Life Science Institute (ILSI), set up in 1978 by a former executive from Coca-Cola, to push for its agenda in the scientific and policy spaces [21]. ILSI also represented tobacco companies in the 1980–90s [22, 23]. ILSI is currently composed of fifteen branches [24], each with a broad range of industry and academic members. The global branch of ILSI is governed by a Board of Trustees that mixes employees from the food industry, including the agribusiness sector (Ajinomoto, PepsiCo, Cargill) and academics [25]. Industry-supported research is also subject to peer-review by the industry itself. ILSI has its own journal, Nutrition Reviews, amongst the most popular journals in nutrition [26]. A recent study found that Nutrition Reviews has the highest proportion of articles with industry involvement (a quarter of all articles from that journal) amongst the top top 10 journals in nutrition [26].

From a public health perspective, somehow, the food industry’s involvement in science and policy is not seen as controversial and harmful as that of the tobacco industry [27, 28]. Some think there is a space for collaboration with that industry, as illustrated in a recent study that tried to build consensus on the interactions between researchers and the food industry [29]. When criticism of the food industry’s involvement in science grew in the 2000s [30–32], ILSI developed guidelines on conflicts of interest (COI) and scientific integrity [20]. These principles call for the involvement of all actors in science, including those from industry actors, and are, not surprisingly, silent on the risks associated with such engagement with industry actors [20, 33]. While there is growing evidence of the food industry’s involvement in science on nutrition and physical activity, little is known of their broader influence on the very principles of scientific integrity.

Our objective was to study the extent of the food industry’s involvement in developing scientific integrity principles, with a case study of ILSI as a key actor in that space.

## Methods

We conducted a qualitative document analysis between February–November 2020, where we triangulated multiple sources of information. We started with initial searches based on an existing scoping review on principles for the interactions between researchers and the food industry. MH conducted searches on the industry’s websites, their social media, and in the Food Industry Documents Library of the University of California, San Francisco [34], an archive containing previously secret internal industry documents. We also used documents from existing freedom of information (FOI) requests made by U.S. Right to Know, a nonprofit investigative public health group. MH and GR independently conducted an initial review of the material for their inclusion against our research objective. MM led the searches on Web of Science and data analysis for all sources of information.

We searched these sources for information related to the development of principles, codes of conduct, frameworks, standards, or other scientific integrity guidelines and responsible research. An analysis of the content and implementation of those principles was beyond the scope of our study.

For the present study, we used the definition of ‘scientific integrity’ from the U.S. National Research Council: “*Integrity characterizes both individual researchers and the institutions in which they work. For individuals, it is an aspect of moral character and experience. For institutions, it is a matter of creating an environment that promotes responsible conduct by embracing standards of excellence, trustworthiness, and lawfulness that inform institutional practices. For the individual scientist, integrity embodies above all a commitment to intellectual honesty and personal responsibility for one’s actions and to a range of practices that characterize responsible research conduct*” [35].

### Initial identification of industry actors

In 2019, MM conducted a backward search, using a recent scoping review by Cullerton et al. and a commentary published in response to that review [36, 37]. The scoping review was purposively selected for our initial searches because it represented the most recent and comprehensive summary of existing principles “*to guide interactions between population health researchers and the food industry*” [36]. The publications identified in the scoping review included work that was funded independently but also work that was supported by the food industry. A response to that review identified additional material from the review sponsored by the food industry [37]. These publications constituted our initial samples of scientific integrity documents developed with industry support (Table 1). This initial sample only included documents where the food industry had direct involvement, through the declarations of interest sections or funding acknowledgments sections or institutions to which the authors were affiliated. By ‘food industry’, we meant any actor along the food supply chain, in the production of raw material, manufacturing, marketing, retailing, and public relations sectors, as well as third parties working on their behalf. We only included those publications that proposed scientific integrity principles, not those broadly discussing the industry’s involvement in science, without providing any guidelines (such as [47, 48]). We also excluded publications on the implementation of such principles at the organizational level, as falling outside the present study’s scope.

**Table 1:** List of food industry actors that have published principles on the interactions between researchers and the food industry and/or on COI in science

Title of publication	Published in a scientific journal?	Publication year	Name of industry actor(s) involved in the publication	Nature of the involvement
Funding food science and nutrition research: financial conflicts and scientific integrity [published in multiple outlets [38–42]]	Yes	2009	**ILSI**	“This paper is the product of a working group on conflict of interest/scientific integrity organized by the North American branch of the International Life Sciences Institute (ILSI North America). It was supported in part by educational grants from Cadbury Adams U.S., LLC, the Coca-Cola Company, ConAgra Foods Inc., General Mills, Kraft Foods, Mars Snackfoods U.S., LLC, PepsiCo Inc., Procter & Gamble, Sara Lee, and Tate & Lyle.” Published in an ILSI journal, Nutrition Reviews.
Ensuring Scientific Integrity: Guidelines for Managing Conflicts [43] [International Union of Food Science and Technology - Scientific Information Bulletin, based on [38]]	No	2012	**ILSI**	“Supported by the ILSI North America Scientific Integrity Working Group”, prepared by the authors of “Funding food science and nutrition research: financial conflicts and scientific integrity” from ILSI
Principles for building public-private partnerships to benefit food safety, nutrition, and health research [44]	Yes	2013	**ILSI**	“This article was commissioned by ILSI North America.” Several authors from ILSI North America, and one from Coca-Cola, and another from Kellogg Company.
Principles and Philosophies for Development of Ongoing Partnerships to Support Food-Health Research [45] [Food for Health Workshop - Canadian Nutrition Society Annual Meeting]	No	2014	**ILSI**	Partnership between the Nutrition Society and ILSI.
Achieving a transparent, actionable framework for public-private partnerships for food and nutrition research [46]	Yes	2015	**ILSI &** **DuPont Nutrition**	Two authors were employees of ILSI, and one author employee of DuPont Nutrition and Health.

With these initial searches, we identified five documents: three scientific articles and two reports. The North American branch of ILSI published four of the five publications, with support from large US-based food manufacturers. Two authors from ILSI also published a fifth article with an author from DuPont Nutrition (DuPont), a dietary supplement manufacturer for the food industry. Therefore, we decided to restrict our following searches to ILSI and DuPont, as they were the only industry actors publishing in the peer-reviewed literature on the topic of scientific integrity.

### Systematic searches on web of science

As a second step, we conducted a literature search to identify further publications on the topic by the ILSI and DuPont, based on the findings of our initial search. On 14 November 2020, MM searched Web of Science Core Collection (Web of Knowledge interface) (our search strategy is available in Additional file 1).

We used the terms (principle* or guid* or ‘codes of conduct’ or framework* or standard* or transparen* or fund*) AND (partner* or integrity or ethic* or inter*) as identified in the titles of publications. We refined the search to publications from ILSI and DuPont, as stated in the declarations of interest sections; funding acknowledgments sections; or institutions to which the authors were affiliated. We had no restriction on the publication time.

All data were extracted from WoS and managed on Mendeley. The publications retrieved from that search were screened for eligibility, based on their titles and abstracts. All data were independently double-screened by A.C. There was no disagreement on the inclusion of documents.

From these systematic searches, no relevant work by DuPont was identified; we, therefore, further restricted our searches for the next steps and focused on ILSI only.

### Industry websites and twitter accounts

MH, with support from EC, identified all websites and Twitter accounts of ILSI Global and its fifteen branches. ILSI’s websites are presented in Additional file 2. MH searched these websites, and social media accounts, for information related to the development of scientific integrity principles. MM then analyzed all data. Our data collection was limited to data available on these websites, and we did not use internet archives to retrieve data that may have been published and then subsequently deleted. In February 2021, ILSI North America transformed into the “Institute for the Advancement of Food and Nutrition Sciences” (IAFNS), a “a non-profit organization that catalyzes science for the benefit of public health” [49]. The URLs for ILSI NA’s webpages in Additional file 2 now redirect to the new IAFNS website. The webpages consulted during data collection could still be consulted using internet acrchives tools like the Wayback Machine [50].

### Archive from industry documents library

Between February and July 2020, MH searched food industry documents in the Food Industry Documents Library of the University of California, San Francisco [34], using standard snowball search methods [51]. Initial keyword search terms included ‘ILSI’, ‘International Life Sciences Institute’, ‘research integrity’, and ‘research transparency’. Twenty-one documents between 2012 and 2018 were located, with most records dated between 2015 and 2017. Documents were screened (MH) and analyzed (MH and MM) for the direct mentioning of information outlining ILSI’s development of scientific integrity principles. Sixteen documents were deemed relevant based on how applicable their contents were to the research objective.

### Documents from existing FOI requests

Additionally, we drew upon nine U.S. federal and state FOI data sets to triangulate our other sources of information: (1) Louisiana State University (Tim Church); (2) University of Colorado (John Peters); (3) Louisiana State University (Peter Katzmarzyk); (4) Texas A&M University (Joanne Lupton); (5) Centers for Disease Control and Prevention (Maureen Culbertson); (6) University of Colorado (James Hill); (7) University of South Carolina (Steven Blair); (8) Louisiana State University (Pennington Biomedical Research Center); (9) U.S. Department of Agriculture (David Klurfeld). U.S. Right to Know filed these FOI requests between 17 July 2015 and 27 December 2017. The requests covered issues regarding sugar sweetened beverages, candy and food companies, and their public relations firms, trade associations, and other allied organizations. The identification of relevant documents for our study was made by GR and his colleague Rebecca Morrison, for their relevance to our research objective.

### Analysis

In November 2020, MM reviewed all data from the sources mentioned above and mapped the actors, timeline of events, and other relevant information related to the food industry’s involvement in the development of scientific integrity principles. In the present manuscript, we present a narrative synthesis of our findings. All authors reviewed the analysis and presentation of findings in the manuscript. We had regular meetings during data collection and analysis, and any disagreement was resolved through discussion within the team. Our existing knowledge informed our analysis of industry influence on science. In the present document, we use the acronym ‘ILSI’ to refer to ILSI North-America, unless otherwise stated. In the results section, we use a code starting with the letter A to refer to our data, all available in Additional file 3.

## Results

Our Web of Science systematic searches yielded 42 publications, 33 of which were excluded as not meeting our inclusion criteria. In addition, one article from 2014, by an author from DuPont, discussed funding by the food industry but did not provide any specific guidelines, so it was excluded [52]. There were eight publications relevant to our research objective on WoS, for our sample of food industry actors. Amongst these eight publications, five were already identified through our initial searches (Table 1 - [38, 44, 46]) with three copies of the same article by ILSI published in different scientific journals simultaneously. The three other studies were also published by ILSI [53–55]. With our searches in internal documents, we found two other publications from the food industry on scientific integrity, both supported by ILSI [56, 57].

In total, we found eight scientific papers from ILSI on scientific integrity, published between 2009 and 2019. In Nov 2020, when writing the current manuscript, these documents were, when combined, cited 364 times (Google Scholar). ILSI also presented its principles in scientific events, reports, and other platforms, as described in Table 1 and below.

Additional file 4 presents a list of authors who published these scientific papers: 63 authors in total, 24 (38%) were from the food industry (as disclosed in the publications). Other authors were from academia, government agencies, and professionals associations, amongst other institutions (see Additional file 4). The majority of the authors were U.S.-based (70%). Five individuals authored four publications (the maximum for a single author), four of them from ILSI and one from academia.

Of note, ILSI promotes these publications on its website, *stating, “ILSI North America has become a leader in scientific integrity and public-private research partnerships for the food and nutrition community. Our work has been published in peer-reviewed journals, endorsed by Federal agencies and professional nutrition and food science societies, and cited broadly throughout the scientific community*” [58].

**Fig. 1:**
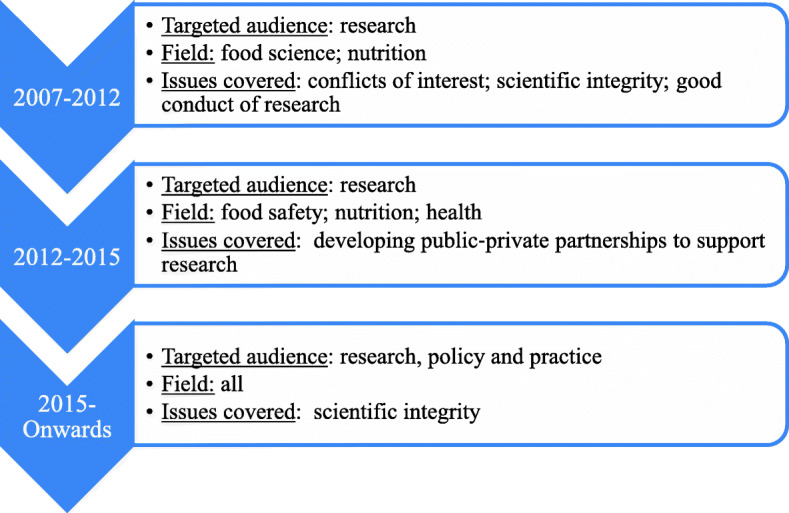
Food industry’s development of scientific integrity principles overtime

Figure 1 summarizes our findings.

In the period 2009–2015, ILSI published articles on conflicts of interest that mainly covered food science, of relevance to food companies, and nutrition, a sub-field of health sciences. During that period, the target audience was researchers. In 2013, a shift occurred, from publishing recommendations on conflicts of interest and the good conduct of research, particularly at the individual and study levels, to proposing guidelines for public-private partnerships (PPP), assuming that PPP would benefit nutrition research. Then, from 2015, ILSI began to target a broader audience, outside academia, such as government agencies and civil society organizations, in its development of scientific integrity principles. At that time, ILSI also started targeting the entire scientific field, and not only the area of nutrition and health.

### 2007–2012: addressing COI in food science and nutrition research

Based on the information we collected, ILSI’s development of scientific integrity principles started in 2007. At that time, the organization “*initiated a program to address COI issues*”, with the rationale that “*despite a wealth of benefits industry sponsored research and science programs have provided, there continues to be significant public debate on the credibility of such support*” [A1]. Over the period 2007–2012, ILSI published COI principles focusing on food science and nutrition research. These publications resulted from different meetings of individuals from the food and agro-industries and academia. At that time, ILSI published on financial conflicts and scientific integrity in food science and nutrition research [38–42].

The first publication is from 2009. The paper originated from a working group at ILSI, the “COI and scientific integrity” working group, and was supported by ten food companies through “*educational grants*” to ILSI [38–42]. Its authors included a mix of employees from ILSI, food companies (Coca-Cola, Kraft, PepsiCo, Cadbury, and Mars), and academics in food science, nutrition, and pediatrics from the U.S. and Canada [38–42]. ILSI said it published this material in six different scientific journals [A2], although we found no trace of the publication in the Journal of Food Science. The article was published in Nutrition Reviews, a journal run by ILSI, the only one of the six journals where the article underwent peer-review. The Academy of Nutrition and Dietetics (formerly American Dietetic Association), who published one copy in its journal, and the American Society for Nutrition (ASN), who published three copies in its American Journal of Clinical Nutrition, Journal of Nutrition, and Nutrition Today, are known to be industry-friendly and receive funding from the food industry [20, 59, 60], which may explain their willingness to publish the paper. The 2009 publication was also adapted, in 2012, into a report of the International Union of Food Science and Technology [38].

In 2011, the ILSI Europe’s Functional Foods Task Force published “*guidelines for the design, conduct and reporting of human intervention studies to evaluate the health benefits of foods*” [53]. The paper named 38 food (including agribusiness) and pharmaceutical companies as members of the taskforce [53]. Amongst the list of authors of the article, six were from the food industry (ILSI, Danone, DuPont (Danisco), Nestlé, and Beneo), three were consultants, and five were academics [53].

In a 2012 letter to ILSI members, Rhona Applebaum, then ILSI’s President and Coca-Cola’s chief health- and science officer, concluded ‘*the program has been highly successful in developing “guiding principles” for industry funding of research*’ [A2]. The success was in the guidelines being “*endorsed by the leadership of three major professional societies. Results of this work have been published in six different peer-reviewed journals and presented at numerous scientific conferences*” [A2]. In that same correspondence, Applebaum sent a list of ILSI’s publications on scientific integrity, where one additional article published in 2011 was included. The latter discussed funding in nutrition research and was published with support from ILSI [56]. The publication was written by four individuals: two from the AND, a consultant, and an academic [56].

### 2012–2015: pushing for public-private partnerships in nutrition research

The period 2012–2013 was a turning point for ILSI, where the discussion on COI in science shifted to the use of science in policy. In her 2012 letter mentioned above to ILSI members, Applebaum stated that there was a “*demand by some that all industry-funded research, whether conducted at contract research organizations or universities, be denied consideration in the formulation of public policy. Furthermore, scientists who have conducted industry-funded research have been barred from serving on public advisory committees*” [A2]. Applebaum, therefore, called ILSI’s food companies members for the “*development of criteria for participation on scientific advisory panels and establishment of appropriate protocols for successful public/private partnerships to advance public health*” [A2]. Food companies were asked to contribute to this task by paying a fee of US$10,000 each [A2].

Therefore, a series of ILSI’s publications on PPP appeared in the scientific literature between 2012 and 2015. In 2012, ILSI’s “*COI and scientific integrity*” working group produced two publications. The first provided suggestions on selecting experts to advise in public policy decision making [57]. The second publication, published in Nutrition Reviews, proposed “*principles for building public-private partnerships to benefit food safety, nutrition, and health research*” [44]. The authors of both publications were a mix of academic experts on the topic, industry employees, and ILSI’s staff.

In January 2014, in a personal communication to prominent physical activity researchers from the US, Applebaum explained that she “*asked ILSI to consider drafting a set of principles on civil discourse in science by scientists similar to what they have done for conflict of interest and public private partnerships*.” She also mentioned: “*There must be a set of guidelines to avoid the current demonizing. They [ILSI] had also been asked to work on principles re selection on gov’t panels since our own U.S. gov’t has raised the issue of working w/ industry as a criterion for non-inclusion*” [A4].

This idea soon translated into concrete action. ILSI first published an article that “*offers counsel on meeting [challenges] in communicating about the work of emerging public-private partnerships*” [61]. This article does not set principles on scientific integrity per se. Still, it is to be understood as part of ILSI’s work in promoting PPP as a means to pursue industry interests.

In 2014, ILSI also started working with third parties on PPP principles, thus accelerating the translation of their work into practice and policy. ILSI proposed to “have a manuscript to share with FDA [U.S. Food and Drug Administration] on best practices for advisory committees”, when the FDA was developing its own COI guidelines [A9].

In parallel, during late 2013, the ASN “*approached ILSI North America to collaborate*” [A109] on activities that would “*stimulate the expansion, accessibility, and acceptance of PPPs by unifying and moving existing principles for food and nutrition research PPPs forward*” [A49]. The ASN convened representatives from the U.S. Department of Agriculture, ASN, Academy of Nutrition and Dietetics, American Heart Association, Centers for Disease Control and Prevention, FDA, Grocery Manufacturers Association, and National Institutes for Health, amongst others [A50]. An individual from the U.S. Department of Agriculture, Klurfeld, and Rowe, a consultant for ILSI, co-chaired a newly formed “*Working Group on Conflict of Interest & Scientific Integrity*” [a name similar to that of ILSI’s “COI and scientific integrity” working group] [A10–1, A14–5]. In 2014, the working group had regular emails, calls, and a face-to-face group meeting in December [later called the “*COI Summit Consortium*”], to agree on a set of PPP principles [A10–5, A29–30]. An ad-hoc steering group was also formed with three USDA staff and a consultant from ILSI, and an ASN staff member [A29].

The whole project was formally led through a “*U.S. government-wide Interagency Committee on Human Nutrition Research*” [A29]. It was formed in 2011 and included a component on PPP, “*in part in response to [a] 2011 Presidential memo directing agencies to develop public-private partnerships in areas of importance to an agency’s mission*” [A29]. In our FOI documents and when justifying the PPP, the ASN made further reference to President Obama, who “*issued a Presidential memorandum in July 2014 encouraging government at all levels to work with private partners on developing infrastructure to lay the foundation for future prosperity*” [A41].

In May 2014, an employee from ILSI sent an email to lead American researchers and employees of federal agencies (U.S. Government Accountability Office and National Institutes for Health), describing the proposed outcome of the newly formed PPP project, a “*summit or collection of major professional societies and federal agencies coming together in support of PPP principles ( …*). *At the conclusion of the summit, the professional societies would agree to a consensus statement on private funding for research and general acceptance of principles for PPPs ( …). it might be helpful for societies who publish journals to have their editors participate in summit*” [A8].

Soon after, in 2015, a peer-reviewed paper outlining the PPP principles in food and nutrition research was published in the Journal of Clinical Nutrition [46] and “*an excerpt of the article appeared in the Journal of the Academy of Nutrition and Dietetics, Journal of Food Science, Nutrition Reviews, and Nutrition Today*” [A66]. In the publication, the authors made clear that the group took “*the ILSI North America published principles as a starting point*” [46], given that “*most reports were not readily accessible in the public domain until, in 2013, a group organized by ( …*) *ILSI North America ( …) published proposed criteria*” [46]. The principles were endorsed by the “*ASN, Academy of Nutrition and Dietetics, American Gastroenterological Association, Institute of Food Technologists, International Association for Food Protection, and ILSI, collectively representing approximately 113,000 professionals*” [A31]. The American Public Health Association declined to endorse the principles but did not justify its decision [A24].

On 16 June 2015, the PPP principles were launched at the National Academy of Sciences. ILSI, in its internal communication, talked of the event and principles as its own: “*There is a meeting today at the National Academies to discuss [PPP] as defined by work that ILSI North America did. ASN and U.S. Department of Agriculture organized the meeting and we expect a number of scientific organizations to adopt the ILSI North America principles*” [A26, A34]. Speakers at that event included the U.S. Department of Agriculture Chief Scientist and Under Secretary, Research, Education, and Economics Dr. Catherine Woteki (keynote address), as well as an ILSI consultant, and an Institute of Medicine Senior Scholar, amongst others [A15, A31].

ILSI and the ASN also had other avenues for disseminating the PPP principles, as detailed in Table 2. The ASN and the Academy of Nutrition and Dietetics were also keen to support a “*Conclave on public-private partnerships*”, where a Declaration would be issued “*to provide a transparent and actionable framework for interested public and private organizations that will minimize external criticism*” [A110].

**Table 2:** list of scientific events were the PPP principles were disseminated

Name of event	Activity undertaken, when information is available
Experimental Biology 2013	An ILSI staff “co-moderated the “Public-Private Partnerships: The Evolving Role of Industry Funding in Nutrition Research””
Academy of Nutrition and Dietetics Food & Nutrition Conference & Expo 2015	
ASN April 2016 Scientific Sessions and Annual Meeting	
3rd and 4th World Conference on Research Integrity	ILSI attended the event, in the 3rd edition, a staff from ILSI participated “in a panel discussion on public-private partnerships and research integrity”.
12th Federation of European Nutrition Societies - European Nutrition Conference	
Congress of the Federation of Latin American Nutrition Societies 2015	ILSI attended and “may be able to distribute copies of article”

Therefore, by having built its own literature on COI principles, scientific integrity, and PPP, and by reaching out to potential allies outside the industry, ILSI naturally became a central and pivotal actor in that discussion.

Hereafter, ILSI took yet another step in disseminating its principles into the scientific and policy spheres, beyond that of nutrition research.

### 2015–2019: beyond nutrition, influencing the very principles of scientific integrity

Hence, after having developed principles for research, and having these principles used to create PPP, ILSI started to evaluate the efforts made by a range of actors to implement scientific integrity principles.

Indeed, in parallel to the work undertaken by the “*U.S. government-wide Interagency Committee on Human Nutrition Research*” working group, ILSI, in 2015, through its own working group, proposed to “*seek a broader group of collaborators than we have previously worked with in order to have a greater impact; ones that have impeccable reputations and are not focused on only one area of science. Possible candidates are: a. American Association for the Advancement of Science; b. Association of Public and Land-grant Universities; c. Association of American Universities; d. The National Academies*” [A80]. ILSI’s working group also suggested that ILSI’s focus “*should be on implementation of these principles/best practices”* [A80]. The group also proposed that when the COI Summit Consortium *“reconvene [s] in two years to reassess the PPP principles ( …) ILSI North America could introduce the principles/best practices for scientific integrity and seek endorsement from the nutrition, food science, and food safety professional societies*” [A80].

As part of that work, in 2017, ILSI set up an “*Assembly on Scientific Integrity*”, whose steering committee included three academics from the University of Illinois, the University of Wisconsin, and Tufts Medical Center, and five employees from Coca Cola, General Mills, Abbott Nutrition, Ocean Spray Cranberries and Biofortis [A79]. The Assembly was made of “*ILSI North America Board of Trustees, all Member Companies of ILSI North America, and the ILSI North America Canadian Advisory Committee*” [A58, A84]. The Assembly was also “*hoping to include government liaisons in the Assembly on Scientific Integrity and it is likely that the ILSI North America Mid-Year meeting in Washington, DC is a better location for government officials to be able to join in-person*” [A107]. In 2017, the budget of the Assembly was US$122,000 [A107].

Then, two authors from ILSI and one from academia, also on the newly formed steering committee and author of other ILSI publications, produced a review of “*efforts by federal agencies, foundations, nonprofit organizations, professional societies, and academia in the United States*” [54]. The review was then translated into a Resource Guide and regularly updated, and similar activity was planned for Canada [A85–6, A98]. Here, the focus was not on food science and nutrition anymore, and the article reported on efforts made by a broad range of institutions like the Centers for Disease Control and Prevention, the Committee on Publication Ethics, the Institute Of Medicine, and the Laura and John Arnold Foundation [54]. The article was published in Critical Reviews in Food Science and Nutrition. ILSI seems to have opened a discussion that is meant to last in that space by inviting readers to “*help keep this document current by pointing out areas that need to be expanded or updated or additional organizations that should be included*” [54].

ILSI’ scientific integrity working group also proposed to *“develop and publish a second paper in collaboration with [the American Association for the Advancement of Science, the Association of Public and Land-grant Universities, and the Association of American Universities] that builds on the first manuscript ( …) to establish the first” rulebook “ on scientific integrity*” [A81]. ILSI convened a meeting in March 2017, where a broad range of actors would discuss the new scientific integrity principles [A86, A101]. The new “*Scientific Integrity Consortium*” was made of “*representatives from four U.S. government agencies, three Canadian government agencies, eleven professional societies, six universities, and three nonprofit scientific organizations*” [A57, A86, A101]. The meeting was organized at the National Academies of Science, Engineering and Medicine as part of the “*Government University Industry Research Roundtable*” [A86, A101], in the same venue used for the launch of the 2015 PPP principles. The group then continued to meet virtually and in-person in 2017 and 2018 [A57, A69, A86]. The “*Scientific Integrity Principles and Best Practices*” were finally published in 2019 in Science and Engineering Ethics [55], reaching a broader audience than merely the nutrition space. ILSI was satisfied that “*the convening of the Scientific Integrity Consortium was a significant step for ILSI North America in building upon our work on scientific integrity and engaging the scientific community beyond the nutrition and food safety community*” [A86]. The long COI section in that publication reports on the many interactions between several of its authors and industry actors [55]. Here again, the Consortium used ILSI’s 2017 findings “*as the basis of the discussion and reconstructed them to form the final set of recommended principles and best practices for scientific integrity*” [55], in combination to some work of the American Society for Microbiology on that topic.

The scientific integrity principles, like those for PPP, were disseminated through different scientific events, in what ILSI called a “*roadshow*” [A104] (see Table 3 for a list of events), with the goal of “*educating attendees (with a focus on young researchers/post docs) on the components of scientific integrity*” [A81]. This time, the audience reached beyond that of nutrition.

**Table 3:** list of scientific events were the scientific integrity principles were disseminated

Name of event	Activity undertaken, when information is available
AND Food & Nutrition Conference & Expo (FNCE) 2018	An oral presentation planned by the ASN and ILSI NA
American Public Health Association 2018 Annual Meeting	An oral presentation, where “supported by the Scientific Integrity Consortium. ILSI North America is an active member of the Consortium.”
American Association for the Advancement of Science 2019 Annual Meeting	
5th and 6th World Conference on Research Integrity	ILSI attended the event
● Association of Public and Land-grant Universities ● Association of American Universities ● American Society of Nutrition- Experimental Biology ● International Association for Food Protection ● Canadian Nutrition Society ● Canadian Child Health Clinician Scientist Program ● Canadian Institute of Food Science and Technology ● Society of Toxicology ● Society for Risk Analysis ● Institute of Food Technologists ● International Union of Food Science and Technology ● International Society for Behavioral Nutrition and Physical Activity ● Committee on Publication Ethics ● World Association of Medical Editors ● International Committee of Medical Journal Editors ● Council for Responsible Nutrition ● Retraction Watch	Other potential venues identified by ILSI

In some of these events, ILSI’s official role in developing the principles was presented as a Consortium member, not its convener [A71]. In October 2017, ILSI shared its Resource Guide directly with the World Conferences on Research Integrity Foundation, who considered using the material for their work [A73, A87]. ILSI, at that time, was seeking to collaborate with the Foundation to further expand its principles globally [A73, A87]. ILSI also planned to develop a training module to implement the new scientific integrity principles and “*a certification program or accreditation ( …) for individuals or organizations to certify their use of the principles and best practices. ( …). It would be beneficial if government agencies would require the certification or accreditation in order to apply for a grant*” [A106].

ILSI is now planning to “*share what we’ve learned with the entire federation of global ILSI entities*” [A67]. ILSI NA’s 2019 Mid-Year Science Program included a presentation on the “*Benefits of More Transparent Research Practices and Bias Reduction Tools*” from a speaker from the Center for Open Science [A59]. ILSI started collaborating with that Center in 2017 [A74, A78]. In 2017 as well, ILSI Argentina formed a new Scientific Integrity Group [A107]. In 2019, the Brazilian branch of ILSI put the question of scientific integrity in the food area as the main topic of its annual congress [A64], with speakers from different Brazilian federal agencies and universities. That same year, an academic from Chile gave a presentation on scientific integrity for the South Andean branch of ILSI [A65].

ILSI continues to try to drive the discussion on scientific integrity in the present COVID-19 pandemic context. In November 2020, ILSI held a webinar where “*invited experts [discussed] some of the challenges that exist for scientists and journals when attempts are made to correct the scientific record - through retractions, corrections or letters/commentaries*”, in response to the “*heightened visibility of retracted publications during the COVID-19 pandemic*” [A68]. The experts in question included some of the authors of the ILSI’s publications presented in our study.

## Discussion

In our study, we found that ILSI is a leading actor, not only in the food industry but more broadly in the scientific community, on the development of scientific integrity standards and principles. Internal and FOI documents revealed the food companies’ motives in developing scientific integrity principles. Food companies have joined forces through ILSI, funded its first activities on COI, and have 38% of the authorship of its scientific integrity publications. We have shown that ILSI built a niche literature, one that would become useful for the food industry, when criticism of its funding of researchers emerged in the U.S. in the mid-2000s [30, 32]. ILSI first focused on COI in food science and nutrition at the individual and study levels, from 2007. Because the literature was scarce on that topic, its publications were used and cited in ILSI’s and others’ further work on COI, scientific integrity and PPP, beyond the field of nutrition and food science. In the past few years, ILSI started to shape the very principles of scientific integrity then and to propose that government agencies, professional associations, non-for-profits, and others, adopt these principles. In the process, ILSI built a reputation in the scientific integrity space. Our study found that ILSI proposed a compulsory certification or accreditation, based on the adoption of its scientific integrity principles, for anyone willing to apply for a research grant. If that were to happen, then ILSI could make it impossible to avoid adhering to its principles. Transparency is often prioritized as per ILSI’s current scientific integrity principles and by government agencies and scientific journals. Transparency should, however, be understood as only one aspect of scientific integrity. It is reasonable to promote the involvement of a broad range of actors in science and to promote good principles for the use of evidence in policy, but ILSI’s work on scientific integrity ignores the risks associated with accepting industry funding [20, 37] and fails to provide guidelines to protect from these risks [19, 37].

It may be that not all individuals and organizations cited in our manuscript were aware that ILSI was founded and is funded by food companies, and that it is food companies that are shaping scientific integrity principles. ILSI, in its publications and communications, presents itself as an independent organization. However, in several of the documents consulted for our study, such as minutes of meetings and emails, and in the scientific publications mentioned here, industry actors were omnipresent. This reveals a state of affairs where the food industry is seen as a legitimate actor in science and policy and where academics see no problem in working with industry actors [28]. In the very process of developing scientific integrity principles, food companies may use their connections with these reputable individuals and organizations to further their influence on science and policy [62, 63].

What we describe here will not be a surprise for ILSI, as they are transparent on these activities, the researchers they fund and indeed promote these principles widely. Some of the information we found during our study was indeed made public. However, internal and FOI documents revealed the true intentions of ILSI behind their development of scientific integrity principles.

This study is novel and builds on several sources to triangulate its findings. Internal industry documents provide a unique behind the scenes look at industry activity and reveal and expose industry behavior rather than speculating about it. This study also has limitations. First, it was beyond the article’s scope to examine all the COI that the individuals identified in our study had with ILSI or other actors in the food industry. Hence, it is highly likely that their relationships extend beyond their authorship on the publications identified here. It is also possible that these authors have published on scientific integrity elsewhere without disclosing their links with ILSI and the food industry. For example, Rowe, a consultant for ILSI on scientific integrity since 2009, published in 2015 a summary of the activities undertaken by ILSI in that space, in one of the chapters, entitled “Principles for Building Public/Private Partnerships to Benefit Public Health”, in the book “Integrity In The Global Research Arena” [64]. In the chapter, there is no reference to the fact that Rowe worked for ILSI and that ISLI has ties with food industry actors. Nevertheless, a broader extent of industry participation would not change the essence of the current findings. Second, this study neither evaluated the content and scientific merit of the scientific integrity principles developed by ILSI and others, nor their implementation. Lastly, our primary focus was ILSI’s work, as our initial searches pointed in that direction, hence potentially leaving out some other work on scientific integrity from other companies and industries, like the pharmaceutical industry. This could be the subject of future investigations.

Our study goes beyond what we know of the food industry’s nutrition and physical activity research funding. It shows that the food industry, like the alcohol and tobacco industries [19], tries to influence science’s very principles, such as scientific integrity and the good conduct of research. Similar to the findings of Ong and Glantz, published 20 years ago on the tobacco industry, the activities described in our paper reflect “*sophisticated public relations campaigns controlled by industry executives ( …) whose aim is to manipulate the standards of scientific proof to serve the corporate interests of their clients*” [14]. Importantly, public health professionals should understand the activities presented here as only one of many practices through which the food industry tries to influence science and policy [15]. This reinforces the call for considering researching the political practices undertaken across industries [65].

## Conclusions

ILSI’s work on scientific integrity, conflicts of interest and public-private partnerships waters down independent work in that space, puts profits before science, and undermines efforts to address undue influence of industry actors on public policy, research, and practice. The industry-established principles have already shaped the evidence on scientific integrity. In the scoping review we identified as a starting point for our searches by Cullerton et al. [36], 14 of the 54 documents included in the review were funded or had involvement of the food industry, despite the clear vested interests that the food industry has in that discussion [37]. Mc Cambridge et al. recently wrote that “*calls for research integrity reflect core values of the research community. They should not be used as instruments to undermine science or to assist harmful industries*” [19]. Therefore, it is crucial that the public health community monitors this work done by ILSI and others and recognizes that seemingly independent organizations like ILSI may represent industry’s interests [15, 19]. This is even more crucial now that ILSI North America transformed itself nto the “Institute for the Advancement of Food and Nutrition Sciences”, a new organization that lacks transparency about its ties with the industry and whose current and future activities remain to be studied [49]. It risks shaping public agencies’ work, which may not be aware of the issues discussed in our paper. The literature we have described here must be understood not to have emerged from within the dietetics or nutrition or even medical professions, but rather from the food industry [14].

## Supplementary Information

**Additional file 1.** Search strategy.

**Additional file 2.** Websites and Twitter accounts of the different branches of ILSI.

**Additional file 3.** Authors on ILSI’s publications on scientific integrity, 2009-2019.

**Additional file 4.** Authors on ILSI’s publications on scientific integrity, 2009-2019.

## Data Availability

All data generated or analyzed during this study are included in this published article [and its supplementary information files].
