# Supplementary material for: Beyond nutrition and physical activity: food industry shaping of the very principles of scientific integrity
Source: Global Health. 2021 Apr 20;17:37. doi: 10.1186/s12992-021-00689-1 (PMC8056799; doi:10.1186/s12992-021-00689-1)
Supplement: Supplementary file 1 — Additional file 1. Search strategy. [file 12992_2021_689_MOESM1_ESM.docx]

**Additional file 1:** Search strategy

TI=((principle* or guid* or ‘codes of conduct’ or framework* or standard* or transparen* or fund*) AND (partner* OR integrity OR ethic* OR inter*))

AND ((OO=(ILSI or DuPont)) OR (FT=(ILSI or DuPont)) OR (FO=(ILSI or DuPont)) OR (DO=(ILSI or DuPont)))
