# Supplementary material for: Beyond nutrition and physical activity: food industry shaping of the very principles of scientific integrity
Source: Global Health. 2021 Apr 20;17:37. doi: 10.1186/s12992-021-00689-1 (PMC8056799; doi:10.1186/s12992-021-00689-1)
Supplement: Supplementary file 2 — Additional file 2. Websites and Twitter accounts of the different branches of ILSI. [file 12992_2021_689_MOESM2_ESM.docx]

## **Additional file 2**: websites and Twitter accounts of the different branches of ILSI

| **Name of the ILSI branch** | **Countries** | **Language(s)** | **Twitter** | **Website** |
| --- | --- | --- | --- | --- |
| Global | All of the Following | English | @ILSI_Global | <https://ilsi.org/> |
| Argentina | Argentina | Spanish | @ILSIArgentina | <http://www.ilsi.org.ar/> |
| Brazil | Brazil | Portuguese | Directed to the @ILSI_Global Twitter account | <https://ilsibrasil.org/> |
| Europe | European Union | English | @ILSI_Europe | <https://ilsi.eu/> |
| Focal Point in China | China | English | Directed to the @ILSI_Global Twitter account | <https://ilsi.org/focalpointinchina/> |
| India | India | English | @ilsiindia | <http://ilsi-india.org/> |
| Japan | Japan | Japanese | Directed to the @ILSI_Global Twitter account | <http://www.ilsijapan.org/> |
| Korea | Korea | Korean and English | Directed to the @ILSI_Global Twitter account | <https://ilsikorea.org/> |
| Mesoamerica | Panama, Nicaragua, Guatemala, Honduras, Trinidad, Tobago, and Costa Rica | Spanish | Directed to the @ILSI_Global Twitter account | <https://ilsimesoamerica.org/> |
| Middle East | No Countries Listed | English | Directed to the @ILSI_Global Twitter account | <https://ilsi.org/middleeast/> |
| North America | English | English | @ILSI_NA | <https://ilsina.org/> |
| South Africa | South Africa | English | Directed to the @ILSI_Global Twitter account | <https://ilsi.org.za/> |
| South Andean | Chile | Spanish | @IlsiSurAndino | <http://www.ilsisurandino.cl/sitio/> |
| Southeast Asia Region | Brunei, Cambodia, Indonesia, Laos, Malaysia, Myanmar, Philippines, Thailand, Singapore, Vietnam, Australia, New Zealand | English | Directed to the @ILSI_Global Twitter account | <https://ilsisea-region.org/> |
| Taiwan | Taiwan | English | Directed to the @ILSI_Global Twitter account | <https://ilsi.org/taiwan/> |
