# Supplementary material for: Beyond nutrition and physical activity: food industry shaping of the very principles of scientific integrity
Source: Global Health. 2021 Apr 20;17:37. doi: 10.1186/s12992-021-00689-1 (PMC8056799; doi:10.1186/s12992-021-00689-1)
Supplement: Supplementary file 4 — Additional file 4. Authors on ILSI’s publications on scientific integrity, 2009-2019. [file 12992_2021_689_MOESM4_ESM.docx]

## Additional File 4: Authors on ILSI’s publications on scientific integrity, 2009-2019

| **Name** | **Affiliation as reported in the publication(s)** | **Disclosed interaction(s) with the food industry, as stated in publications** |
| --- | --- | --- |
| 1. Sylvia Rowe | SR Strategy, USA  Then Tufts Friedman School & University of Massachusetts at Amherst, USA | Consultant - ILSI North America |
| 1. Nick Alexander | SR Strategy, USA | Consultant - ILSI North America |
| 1. Fergus Clydesdale | The University of Massachusetts at Amherst, USA | Trustee - ILSI North America and  the International Food Information Council “Scientific advisor to several companies in the food and nutrition industries” |
| 1. Rhona Applebaum | Coca-Cola Company, USA  ILSI North America, USA | Employee |
| 1. Stephanie Atkinson | McMaster University, Canada |  |
| 1. Richard Black | Kraft, USA | Employee |
| 1. Johanna Dwyer | New England Medical Center, USA  Then Tufts Medical Center, USA |  |
| 1. Eric Hentges | ILSI North America, USA | Employee |
| 1. Nancy Higley | PepsiCo, USA  Then Kerry Ingredients and Flavours, USA | Employee |
| 1. Michael Lefevre | Utah State University, USA |  |
| 1. Joanne Lupton | College Station, USA |  |
| 1. Sanford Miller | University of Maryland Center for Food, USA |  |
| 1. Doris Tancredi | Cadbury Adams, USA | Employee |
| 1. Connie Weaver | Purdue University, USA |  |
| 1. Catherine Woteki | U.S. Department of Agriculture, Research, Education, and Economics, USA [Former]  Then Mars, Inc., USA [Former]  Then Iowa State University, USA | Employee |
| 1. Elaine Wedral | ILSI North America, USA | Employee |
| 1. Robert W.Welch | University of Ulster, U.K. | “made presentations sponsored by food companies, and has been a member of  research teams that have carried out projects funded wholly or partly by food companies” |
| 1. Jean-Michel Antoine | Danone, France | Employee |
| 1. Jean-Louis Berta | Consultant, France |  |
| 1. Achim Bub | Federal Research Centre for Nutrition and Food, Germany |  |
| 1. Jan de Vries | De Vries Nutrition Solutions, The Netherlands | Employee |
| 1. Francisco Guarner | Hospital General Vall d'Hebron, Spain |  |
| 1. Oliver Hasselwander | Danisco/DuPont, U.K. | Employee |
| 1. Henk Hendriks | TNO Quality of Life, The Netherlands |  |
| 1. Martin Jäkel | Unilever, The Netherlands | Employee |
| 1. Berthold V. Koletzko | Dr. Von Hauner Children's Hospital, University of Munich Medical Centre, Germany |  |
| 1. Chris C. Patterson | Queen's University Belfast, UK |  |
| 1. Myriam Richelle | Nestlé, Switzerland | Employee |
| 1. Maria Skarp | ILSI Europe, Belgium | Employee |
| 1. Stephan Theis | Beneo, Germany | Employee |
| 1. Stéphane Vidry | ILSI Europe, Belgium | Employee |
| 1. Jayne V. Woodside | Queen's University Belfast, UK |  |
| 1. Esther F. Myers | Academy of Nutrition and Dietetics, USA |  |
| 1. J. Scott Parrott | University of Medicine and Dentistry of New Jersey, USA |  |
| 1. Deborah S. Cummins | Academy of Nutrition and Dietetics, USA |  |
| 1. Patricia Splett | Splett and Associates, USA |  |
| 1. Christa Drew | Center for Nonviolent Solutions, USA |  |
| 1. Elizabeth Westring | General Mills, USA | Employee |
| 1. Alison Kretser | ILSI North America, USA | Employee |
| 1. Robert Steele | Pennsylvania State University, USA [retired] | Former trustee - ILSI North America |
| 1. Molly Kretsch | US Department of Agriculture, USA |  |
| 1. Juan Navia | McNeil Nutritionals, USA | Employee |
| 1. Ashley Jarvis | ILSI North America, USA | Employee |
| 1. Ken Falci | Ken Falci Consulting, USA  Kellogg Company, USA [formerly] | Employee |
| 1. Robert E Brackett | Illinois Institute of Technology, USA | Director - Illinois Institute of Technology Institute for Food Safety and Health  & receives financial support and grant funding from various member companies  & member - ConAgra Food Safety Council and the FMC Advisory Council |
| 1. Britt Burton-Freeman | Illinois Institute of Technology, USA |  |
| 1. David M Klurfeld | USDA Agricultural Research Service, USA |  |
| 1. Linda D Meyers | American Society for Nutrition, USA |  |
| 1. Ratna Mukherjea | DuPont, USA | Employee |
| 1. Sarah Ohlhorst | American Society for Nutrition, USA |  |
| 1. Delia Murphy | ILSI North America, USA | Employee |
| 1. Stefano Bertuzzi | American Society for Microbiology, USA |  |
| 1. Todd Abraham | ILSI Global Board of Trustees, USA [former] | Former board member - ILSI |
| 1. David B. Allison | Indiana University, USA |  |
| 1. Kathryn J. Boor | Cornell University, USA |  |
| 1. Andrea Grantham | Canadian Nutrition Society, Canada |  |
| 1. Linda J. Harris | International Association for Food Protection, USA  and the University of California, USA |  |
| 1. Rachelle Hollander | National Academies of Sciences, Engineering, and Medicine, USA [former]  Then National Academy of Engineering, Center for Engineering Ethics and Society, USA |  |
| 1. Chavonda Jacobs-Young | U.S. Department of Agriculture, USA |  |
| 1. Sarah Rovito | Association of Public and Land-Grant Universities, USA |  |
| 1. Dorothea Vafiadis | National Council on Aging, USA &  American Heart Association, USA |  |
| 1. Jessica Wyndham | American Association for the Advancement of Science, USA |  |
| 1. Rickey Yada | University of British Columbia, Canada |  |
